# Early Diagnosis of Pancreatic Cancer: Clinical Premonitions, Timely Precursor Detection and Increased Curative-Intent Surgery

**DOI:** 10.1177/10732748231154711

**Published:** 2023-01-31

**Authors:** Kjetil Søreide, Warsan Ismail, Marcus Roalsø, Jacob Ghotbi, Claudia Zaharia

**Affiliations:** 1 Department of Gastrointestinal Surgery, HPB unit, 60496 Stavanger University Hospital, Stavanger, Norway; 2 Department of Clinical Medicine, University of Bergen, Bergen, Norway; 3 Gastrointestinal Translational Research Group, Laboratory for Molecular Medicine, 60496 Stavanger University Hospital, Stavanger, Norway; 4 Department of Quality and Health Technology, 60496 University of Stavanger, Stavanger, Norway; 5 Department of Pathology, 60496 Stavanger University Hospital, Stavanger, Norway

**Keywords:** screening, early detection, biomarker, radiology, diagnosis, curative surgery, prevention, early diagnosis, liquid biopsy

## Abstract

**Background:**

The overall poor prognosis in pancreatic cancer is related to late clinical detection. Early diagnosis remains a considerable challenge in pancreatic cancer. Unfortunately, the onset of clinical symptoms in patients usually indicate advanced disease or presence of metastasis.

**Analysis and Results:**

Currently, there are no designated *diagnostic* or *screening tests* for pancreatic cancer in clinical use. Thus, identifying risk groups, preclinical risk factors or surveillance strategies to facilitate early detection is a target for ongoing research. Hereditary genetic syndromes are a obvious, but small group at risk, and warrants close surveillance as suggested by society guidelines. Screening for pancreatic cancer in asymptomatic individuals is currently associated with the risk of false positive tests and, thus, risk of harms that outweigh benefits. The promise of cancer biomarkers and use of ‘omics’ technology (genomic, transcriptomics, metabolomics etc.) has yet to see a clinical breakthrough. Several proposed biomarker studies for early cancer detection lack external validation or, when externally validated, have shown considerably lower accuracy than in the original data. Biopsies or tissues are often taken at the time of diagnosis in research studies, hence invalidating the value of a time-dependent lag of the biomarker to detect a pre-clinical, asymptomatic yet operable cancer. New technologies will be essential for early diagnosis, with emerging data from image-based radiomics approaches, artificial intelligence and machine learning suggesting avenues for improved detection.

**Conclusions:**

Early detection may come from analytics of various body fluids (eg ‘liquid biopsies’ from blood or urine). In this review we present some the technological platforms that are explored for their ability to detect pancreatic cancer, some of which may eventually change the prospects and outcomes of patients with pancreatic cancer.

## Introduction

**Figure 1.**
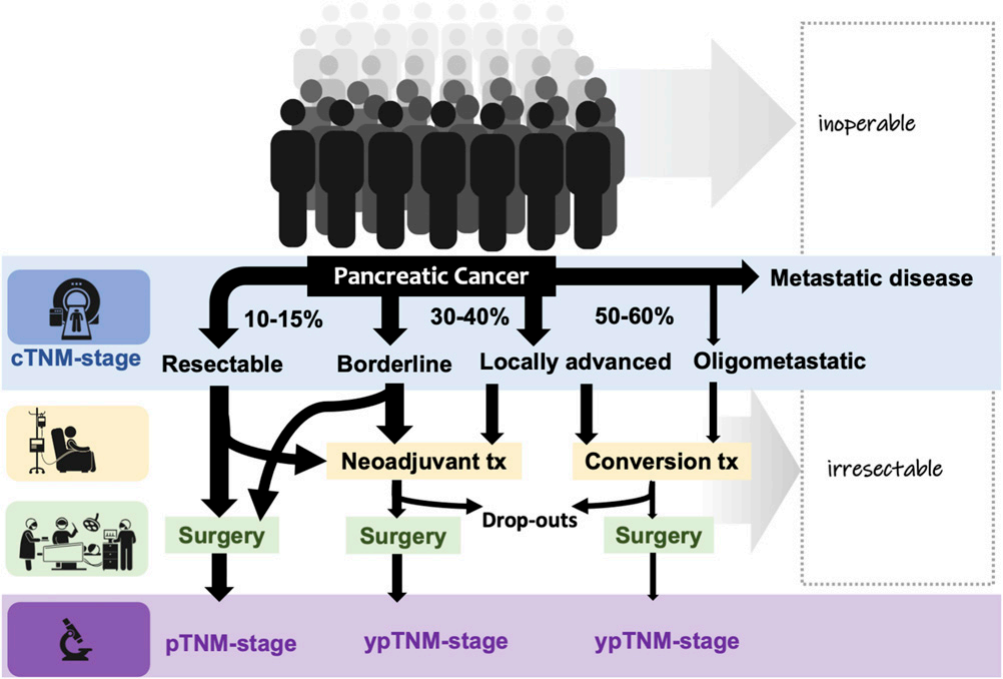
Proportion of patients presenting for potential curative treatment. Legend: Any given patient may be deemed inoperable at time of diagnosis or irresectable through clinical (image-based) staging. Definitions for borderline/locally advanced cancers are floating, with variation in management. More effective systemic therapy (eg FOLFIRINOX) is increasingly introduced in the pre-operative setting, with more resections offered after therapy, possibly influencing the pathological TNM-staging and interpretation of its prognostic role. Better predictive and prognostic biomarkers of cancer biology are needed. Reproduced with permission from Roalsø et al. Copyright © 2020 The Author(s). Published by Elsevier Ltd. All rights reserved.

Pancreatic cancer is increasing in incidence and will soon become a major cause of cancer-related deaths in several parts of the world.^1^ Pancreatic cancers are typically diagnosed at a time when the patients have developed symptoms, usually indicating locally unresectable disease and/or metastasis.^2,3^ Currently, only 15-20% are diagnosed at a stage when curative surgery may be considered (Figure 1). Unfortunately, symptoms are in general vague and unspecific in most patients. Of concern is an increase in the early-onset rates of pancreatic cancers reported from several countries.^4-7^ Predictions suggest that pancreatic cancer will become 1 of the most common causes to cancer-related deaths in most Western countries within a few years. Hence, a more timely diagnosis and more efficient therapy is urgently needed.^8^

**Figure 2.**
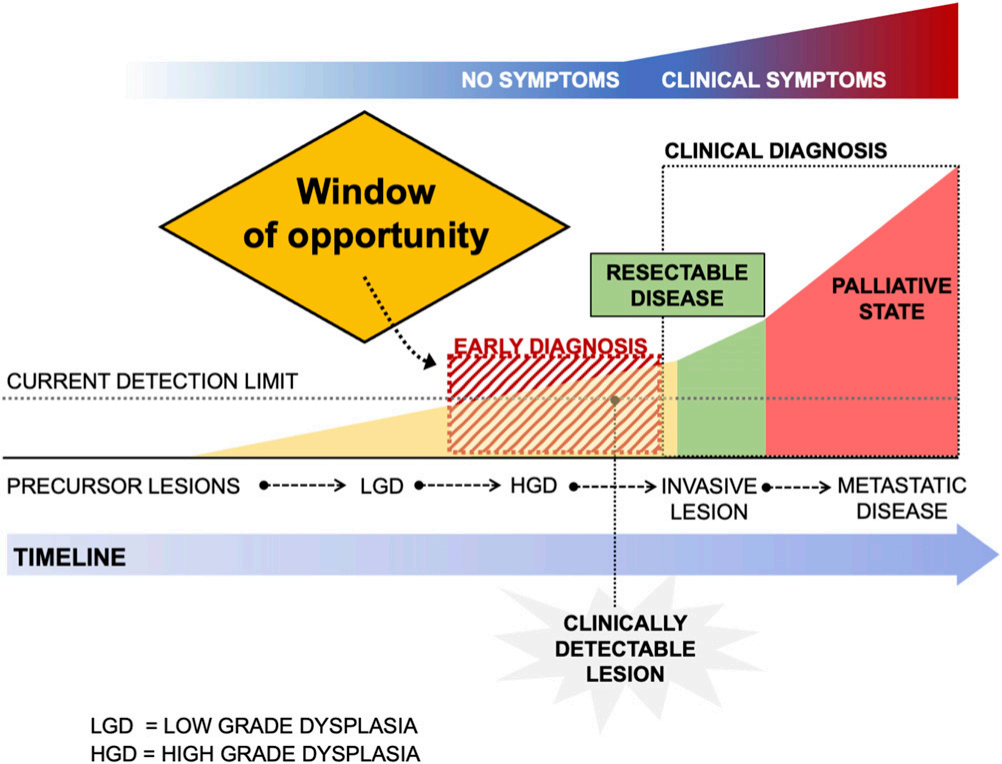
Window of opportunity for early detection of pancreatic cancer. Legend: The early detection of resectable disease or precursor lesions requires earlier detection at a time when no symptoms are present, yet biological signals (eg imaging, blood tests, biomarkers) are present for detection. Detection and treatment of high-grade dysplasia (HGD) before invasive cancer may provide cure (yellow zone); detection of early-stage cancer (green zone) may improve survival and cure rates.

The idea behind the effect of screening is that early detection of disease in an asymptomatic or precursor stage will allow for timely treatment and hence improve prognosis.^9^ The criteria and principles set out by the World Health Organization^10^ (WHO) for justifying public screening programmes include a list of 10 points, including the need to address an important health problem; availability of accepted treatment for the condition; recognizable latent or symptomatic stages of the disease; suitable test or examinations to detect the disease; the test should be acceptable to the population; proper understanding of the natural history of the disease should be available; cost-efficiency of test and economic burden to medical care should be available; agreed policy to treatment, to mention some of the scientific principles.^9,10^ Unfortunately, pancreatic cancer is not suited for population screening given the overall low incidence of the disease and the current lack of accurate, inexpensive and non-invasive screening tests. Hence, population-based screening for pancreatic cancer is currently not recommended and should be avoided. However, there is a dire need to identify groups in the general population of asymptomatic individuals that are at a higher risk for developing pancreatic cancer. The precursor stages have been defined and should allow intervening with preventive strategies or early surgery by early detection of pre-symptomatic, non-invasive disease in a “window of opportunity” (Figure 2).

In the future, defining at-risk groups may be needed for cohort studies of screening, for studies of early diagnosis or, for preventive intervention strategies. In this article, we will discuss some emerging areas raising an opportunity for earlier detection of pancreatic cancer.

## The Challenge With Early Cancer Detection and Early Cancer Stages

All cancers have risk factors attributed to lifetime exposures that may trigger tumorigenesis and enhance malignant progression. Pancreatic cancer develops through defined cellular and molecular pathways, with well-described precursors such as Pancreatic Intraepithelial Neoplasia (PanIN) and cystic mucinous precursors Mucinous Cystic Neoplasms (MCNs) and Intraductal Papillary Mucinous Neoplasms (IPMNs), harbouring specific associated genetic characteristics.^11^ PanIN lesions are microscopic, typically found in resected specimens, often for other reasons and generally cannot be detected on preoperative imaging. PanIN lesions precede any development of clinically detectable disease. They represent part of a multistep tumor progression model to invasive ductal adenocarcinoma in which increasing morphological grades of dysplasia are accompanied by accumulation of various genetic alterations.^12^ Given its microscopic nature, PanIN is currently not a target for screening as it is mostly a finding on histopathology. Hence, these premalignant lesions are usually identified either through intense surveillance of populations at particularly high risk, such as those in surveillance programs.^13^ In high-risk individuals, high grade PanIN is frequently multifocal and often associated with lobulocentric atrophy that has been suggested for possible detection on EUS, suggesting indirectly a potential screening tool in this particular group of patients.^14,15^ However, most premalignant lesions are detected as incidental pancreatic cysts on conventional imaging.^16^

Early-onset cancers has been called an emerging global epidemic, also for pancreatic cancer.^17^ Also, more patients are diagnosed with early-stage cancers (stage IA), suggesting that closer surveillance of high-risk groups may contribute to an earlier diagnosis.^5^ However, the relative contribution is small, with less than 1% being diagnosed as “early cancers” in the beginning of the study period only to rise to less than 3% at the end.^5^ This is in parallel to a study from England, showing that stage I made up less than 1% of all resected pancreatic cancers, and stage II made up less than 2%.^6^ A similar rate was corroborated in a multi-center Japanese cohort, with less than 1% and 3% being stage I and II, respectively.^7^ As such, early-stage cancers make up a very little part of all pancreatic cancers. Further, a screening test would require a very high diagnostic specificity (>95%) to avoid generating too many false-positive tests.^18^ Therefore, pancreatic cancer is not included for screening in the general population in most countries.^19-21^

## The Challenge With Cancer Screening

**Figure 3.**
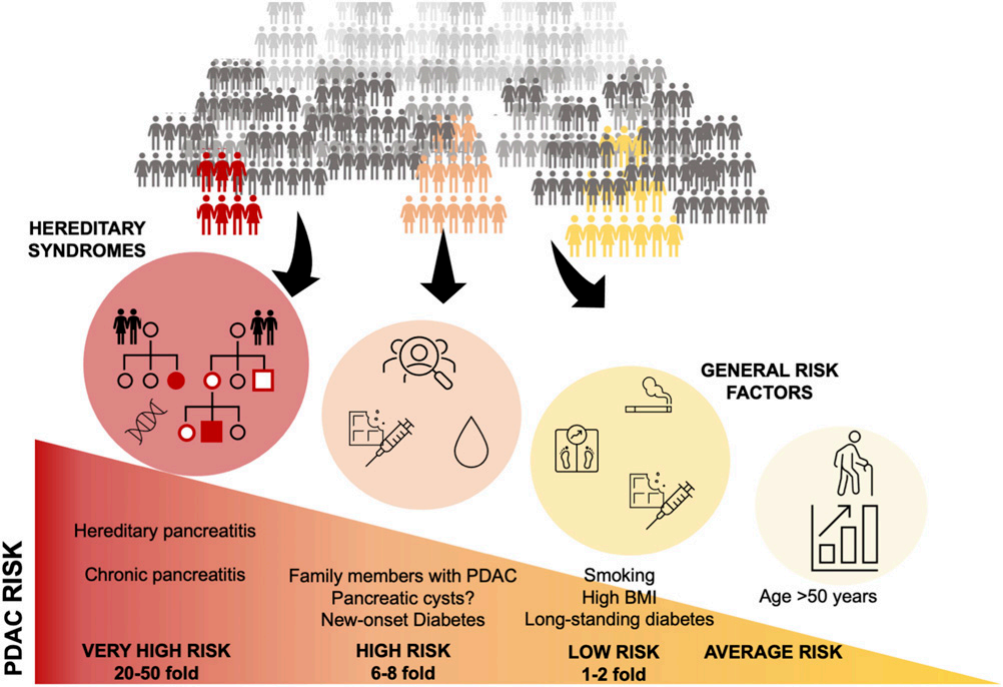
Relative risk for pancreatic cancer in the population. Legend: Highest risk is found in hereditary genetic syndromes, yet the majority present without specific risk factors.

For screening of a disease to be effective, the disease should be diagnosed at an early, asymptomatic stage when cure is possible, but for pancreatic cancer this is a rare event in clinical practice.^22^ The prerequisite for any screening program,^9,10^ is having a patient population with a high enough prevalence of the disease that 1 is looking for, as even a good test will suffer from a low prevalence and result in low yield, ie low positive predictive value. As such, the first challenge should be identifying high-risk groups (Figure 3).

Even in early-stage cancers, only about 1 in 5 may present without any symptoms.^7^ The consensus is that widespread population-based screening for pancreatic cancer in the general population is neither practicable nor indicated in most countries.^20,21,23,24^ One report concluded that screening for pancreatic cancer would not improve disease-specific survival based on the rapid progression of the disease; the overall benefits was estimated to be small at best; and, that screening would be associated by a modest risk of harms.^25,26^ Consequently, screening is not supported in most guidelines.^27,28^

An ideal test for early detection (and, prevention) would include a sensitive, accurate serum marker to detect asymptomatic cancers that are otherwise clinically and radiographically undetectable. Additionally, the marker should allow isolation of the organ involved and, since the lesion is too small to detect, be able to be treated with natural products (eg dietary compounds, or food products) to prevent growth and for the marker to become undetectable. The sensitivity of a biomarker-based screening test will need to be much higher for cancers with a modest public health burden than for those with larger burdens.^29^ One important reason for this is that small changes in the sensitivity of any biomarker (alone; as a panel, or; as an imaging modality) applied for screening purposes can have modest or enormous impacts on system-wide costs per cancer detected,^30^ depending on the prevalence of the disease being screened.^31,32^ Currently, such a screening test that satisfies all criteria is not available for pancreatic cancer.

### Screening of High-Risk Individuals

Patients with high risk (>5% life-time risk) of PDAC are currently offered screening in certain programmes. Certain risk-groups with hereditary syndromes (Table 1) and familial pancreatic cancer are included in ongoing programs for early detection.^33^ Persons with pancreatic cystic lesions is another risk group,^16^ for which some need surveillance while others may need resection.

**Table 1.** Hereditary Genetic or Cancer Syndroms and PDAC Risk.

Syndrome	Mutation	Lifetime risk %
Peutz-Jeghers syndrome	STK11 (LKB1)	11-32
Hereditary pancreatitis	PRSS1	25-40
FAMMM	P16INK4A/CDKN2A	17
Lynch syndrome (HNPCC)	MLH1, MSH2, MSH6, PMS2, EPCAM	8.6
FAP	APC	1.7
Cystic fibrosis	CFTR	<5
HBOC syndrome	BRCA1, BRCA2/FANCD1, PALB2/FANCN, FANCC, FANCG	Increased
Ataxia telangiectasia	ATM	Increased

FAMMM, Familial atypical multiple mole melanoma syndrome; FAP, familial adenomatous polyposis; FDR, first degree relative; HBOC, hereditary breast and ovarian cancer; HNPCC, hereditary nonpolyposis colorectal cancer; PDAC, pancreatic ductal adenocarcinoma.

A systematic review^34^ of prospective cohort studies (including those with more than 20 patients) of asymptomatic adults determined to be at high-risk of pancreatic cancer (lifetime risk >5%, including specific genetic-associated conditions) who were screened by endoscopic ultrasound (EUS) and/or magnetic resonance imaging (MRI) to detect pancreatic lesions. The investigators^34^ found 19 studies with a total of 7085 individuals at high risk for pancreatic cancer. Of these, 1660 patients were evaluated by EUS and/or MRI. Fifty-nine high-risk lesions were identified (43 adenocarcinomas, of which 28 during the initial exam and 15 during follow-up surveillance) and 257 patients had pancreatic surgery. Based on the meta-analysis,^34^ the overall diagnostic yield screening for high-risk pancreatic lesions was .74 (95% CI, .33-1.14), with moderate heterogeneity among studies. The ‘number needed to screen’ to identify 1 patient with a high-risk lesion was 135 persons (95% CI, 88-303) per detected high-risk lesion. The diagnostic yield was similar for patients with different genetic features that increased risk, and whether patients were screened by EUS or MRI.^34^ Hence, the screening yield, even in high-risk populations, is currently labour-intensive with a modest outcome on early detection rates and opportunity for intervention. However, it is expected to see improvements as technology and tools develop and population at-risk definitions are refined.

## Population at Risk for Pancreatic Cancer: Emerging Data

Most patients with pancreatic cancer are diagnosed after presentation of symptoms (Figure 1 and 2) with some higher-risk groups undergoing surveillance.^35,36^ Unfortunately, the clinical symptoms occur late. Weight loss and/or silent jaundice may be robust indicators for an underlying cancer that warrant referral and work up,^37^ but are usually associated with already advanced disease or metastases. Notably, the most common risk factors (including age, smoking, obesity) are too generic and do not warrant screening per se. Hence, most patients are unfortunately diagnosed when cure is no longer possible (Figure 1).

**Figure 4.**
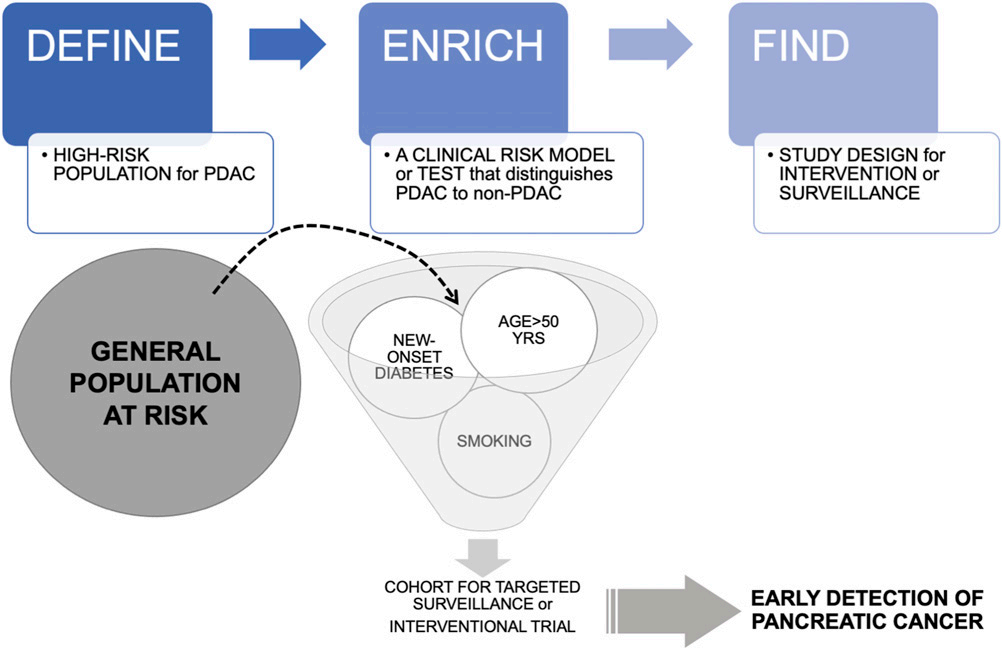
The Define-Enrich-Find strategy for early detection of pancreatic cancer. Legend: Screening for sporadic PDAC in the average risk general population is considered unrealistic because of the low incidence. An alternative to screening is a proposed DEF (Define, Enrich, Find) strategy that allows PDAC surveillance in a subset of higher risk asymptomatic patients where it might be most beneficial. New-onset diabetes or pancreatic cysts may be such targeted populations.

Thus, there is a need to narrow the sieve through which subjects with a particular risk are enriched (Figure 4), so as to increase screening accuracy and cost-effectiveness. One way would be to narrow down the population at-risk going through the screening system (Figure 3). A specific risk group of increasing attention is subjects >50 years of age with new-onset diabetes – a population with the highest risk for sporadic PDAC.^38^ However, even in this scenario with an estimated pancreatic cancer prevalence of .8% the risk-benefit scenario is complex even with an assumed very sensitive and specific test.^18^ Indeed, identifying robust, valid risk factors for appropriate screening and early detection of pancreatic cancer is challenging, as demonstrated in several epidemiological models.^39-42^

## Cross-Section Imaging for Detection

Imaging is the current diagnostic reference standard for pancreatic lesions. Imaging consists of endoscopic ultrasound (EUS), computed tomography (CT) and magnetic resonance imaging (MRI). Each have benefits and disadvantages, and all are equally accurate in diagnosing pancreatic cancer,^43^ together with transabdominal ultrasound and contrast-enhanced ultrasound.^44^ However, no imaging modality is practical as stand-alone screening tool in individuals at regular risk for pancreatic cancer. Notably, imaging has detection limits regarding size. Cysts are the only visible precursor lesion, as PanINs are not detected on standard imaging. However, pancreatic cystic precursors such as IPMNs or premalignant mucinous cystic lesions are detectable with imaging studies.^16,45^ An increasing number of individuals are diagnosed with incidental pancreatic cysts.^16,46^ Notably, the guidelines for surveillance or resection are conflicting, with considerable variation in the recommendations of observation vs resection.^16,46-50^ However, individuals with cystic lesions represent a defined risk population for exploring biomarkers to assess risk and define progression from precursor to invasive cancer.^51,52^

## Biomarkers for Early Detection of PDAC

Biomarkers have yet to make an impact on early diagnosis for pancreatic cancer, even if there is no lack of suggested candidate markers in the available literature.^53-62^ The *Alliance of Pancreatic Cancer Consortia for Biomarkers for Early Detection* provided a common platform, listed the resources necessary for validation and, named the available markers felt to be promising for further pursuit.^63^ None of the biomarkers were ready for a large-scale trial for biomarker validation.

In systematic review reports, several markers have been labelled as promising, yet remain under investigation for clinical utility. Extracellular vesicle (including microRNAs and others) as biomarkers have been scrutinized,^60^ yet technological difficulties and standardization needs to be overcome before translation into clinical use.

One study has explored the utility of early elevation of CA 19-9 as an “anchor test” together with other biomarkers, to identify risk of early pancreatic cancer up to 5 years prior to diagnosis.^64,65^ This is promising, giving the ubiquitous use of CA 19-9. However, about 10% in the population will be Lewis-antibody negative, and hence not express CA 19-9 at all even if cancer is present. Thus, a more universally expressed marker with sufficient sensitivity may be needed.

## New-Onset Diabetes, Glucose Intolerance and Metabolic Alterations

A strong correlation to risk of developing PDAC is associated with reduced blood glucose tolerance and new onset diabetes.^66^ In 1 meta-analysis, with every .56 mmol/L increase in fasting blood glucose there was an associated with a 14% increase in the rate of PDAC.^67^ In a model (Enriching New-Onset Diabetes for Pancreatic Cancer; ENPAC) based on changes in weight, change in blood glucose, and age at onset of diabetes, the investigators found persons with a score ≥3 to have 80% sensitivity and specificity for developing PDAC.^42^ While needing validation, such risk scores could improve risk-stratification to improve the diagnostic yield by use of a screening test or modality.

Blood glucose alteration is but 1 among several metabolic changes that may follow the progression or even be caused by pancreatic cancer.^35^ While fasting blood glucose may be a target based on the PDAC specific mechanisms to increased blood glucose, several other metabolic alterations occur in PDAC, involving muscle mass, lipids and protein synthesis.^68-73^

Higher levels of branched-chain amino acids have been found to occur years before diagnosis of PDAC in several studies, suggesting these to be metabolomic biomarkers for future PDAC risk.^72,74,75^ In 1 study,^75^ elevated plasma levels of branched-chain amino acids (BCAAs) are associated with a greater than 2-fold increased risk of future pancreatic cancer diagnosis. This increased risk was independent of other, known predisposing factors. The strongest association was observed among subjects with samples collected 2 to 5 years before diagnosis of PDAC.

In an attempt at validation of the findings, a European cohort data (from Norway, Finland, Estonia and the Netherlands) did not support the branched-chain amino acids identified earlier in several US cohorts as potential biomarkers for pancreatic cancer.^76^ The European cohorts rather identified glutamine and histidine as potential biomarkers of interest. However, they investigators concluded that the study did not yield metabolomic biomarkers with sufficient predictive value to be clinically useful as a prognostic biomarkers.

One general problem with several of the proposed biomarkers is that the sample is collected at the time of PDAC diagnosis (or, even later after diagnosis) which may not correctly reflect the metabolomic profile year before a diagnosis is made. Similar experience has been made with other types of serum markers, including microRNA in serum.^77^

## Further Developments and Novel Technology

Novel approaches are investigating non-invasive biomarkers that can be easily accessed or monitored, of which some will be briefly mentioned here. The attractive principle for most biomarkers for early detection would be a test that is a non-invasive, repeatable test which would allow for early detection of resectable PDAC with potential for cure, or better, prevention by operation of high-grade dysplasia not yet transformed into invasive cancer.^36^ Some tests have been proposed for use by sampling saliva^59,78-81^ or urine^52,61,82-85^ for detection of pancreatic cancer. However, these technologies and their accuracy needs further refinement before being introduced as useful clinical tests. Also, the various use of biosensors,^86,87^ although attractive, have yet to see a development that is near clinical implication. Hence, we have focused on the role of test already in routine use, such as the expanded use of conventional imaging information (radiomics), the evolving role of sampling pancreatic juice and analyses, and the emerging role of liquid biopsies and markers in blood.

### Cross-Sectional Abdominal Imaging Tools and Radiomics

Radiomics is a sub-field of computer vision analysis. The core premise of radiomics is that the differences in size, shape, texture, and greyness of a tumor contoured from a radiological image can reflect the variations in histological phenotype and genotype of the tumor.^88^ Briefly explained, various radiological images (typically CT or MRI scans) can be converted into mineable data through which high-throughput extraction of quantitative features can be done by computers. The extracted data can then be combined with clinical features to generate a diagnostic or prognostic model for cancer or, even by means of adding artificial intelligence or machine learning allow for early detection of cancer.^89-93^ However, there is a need to harmonize data towards a common standard.^94^

Current studies on quantitative imaging biomarkers in pancreatic cancer are hampered by small sample sizes, together with a lack of standardization in image pre-processing and acquisition protocols, external validation, and the substantial heterogeneity in features analyzed, making it hard to compare data sets.^95^ Thus, radiomics is currently not recommended for routine clinical practice. No commercially available radiomics solution exist for pancreatic tumors, albeit progress is being made in automating image segmentation, lesion characterization and cancer detection.^96^ Machine learning is a technique for analyzing and predicting by learning from sample data, finding patterns in it, and applying it to new data.^90^ Early detection of pancreatic cancer is challenging due to overlapping imaging features with benign lesions, though quantitative imaging has helped differentiate autoimmune pancreatitis from PDAC with an accuracy of 95.2%,^97^ and all PDAC cases were correctly classified when compared to healthy pancreata,^98^ both studies showcasing radiomics as useful in differentiating pancreatic disease states. In addition, a proof-of-concept study utilizing clinical data, miRNAs and radiomics in 38 surgically resected, pathologically confirmed IPMN cases, managed to predict malignant IPMNs superior to conventional models.^99^ Of significance, here radiomics helped identify the true negatives from otherwise false positives, and correctly classified a true positive, which was false negative using conventional imaging results; findings which can simultaneously lead to a reduction in pancreatic resections, and correctly identify patients in need of surgery. Further, non-invasive insights have been found utilizing radiomics regarding drug sensitivity, tumor subtypes, treatment response and clinical stratification, which ultimately can help guide patient care.^100-102^ Radiomics with machine learning has proposed to be able to detect PDAC up to 2 years prior to diagnois in 1 study.^103^

### Pancreatic Juice and Cyst Fluids

Detection of biomarkers in pancreatic juice have been explored across several clinical settings, including for high-risk subjects with familial risk or for patients with pancreatic cystic lesions. Both genomic, metabolomic and proteomic biomarkers have been explored.^88,104-107^ Studies on early cancer detection through analysis of pancreatic juice, including brush cytology during ERCP, show sensitivity ranging from 21.3% to 63.6% and specificity of 94% to 100%.^108^ However, pancreatic juice analysis could be affected by the position and size of the catheter and in addition, patients may suffer from frequent complications, such as ERCP-associated pancreatitis.^109,110^

Sometimes it is not possible to obtain a diagnostic sample on EUS or ERCP, even if imaging suggests a lesion or suspicious finding. In this setting, some investigators have used a technique called *serial pancreatic juice aspiration cytologic examination* (SPACE) for the diagnosis.^111-114^ The approach is not universally available, and early experience comes mainly from Japan, but may represent a useful diagnostic method in select cases for early-stage pancreatic cancers, such as carcinoma in situ that are difficult to diagnose by endoscopic ultrasound-guided fine needle aspiration (EUS-FNA).^113^ The method is initiated via ERCP, whereby an endoscopically placed nasopancreatic drainage allows for serial measurements of pancreatic juice for cytology, or ‘liquid biopsy’.^114^ Of note, the method carries the risk of post-ERCP pancreatitis.

High concentration of carcinoembryonic antigen (CEA) in pancreatic cyst fluid is reflective of a mucinous cystic precursor and associated with 57-79% sensitivity. Cytologic examination of cyst fluid regarded as an enhancement of EUS’ utility in identifying cystic neoplastic precursors, reaches high specificity rates, but the technique is hampered by low cellularity and therefore the sensitivity varies from 25 to 88%. Genomic alterations revealed by *next generation sequencing* (NGS) of exfoliated epithelium in cyst fluid correlate with mutational profiles of the major mucinous pancreatic cysts and those that progressed to invasive carcinoma. For example, the detection of KRAS mutations in pancreatic cyst fluid by NGS shows a 76%-89% sensitivity and 96%-100% specificity for IPMNs and MCNs. Mutational analysis of pancreatic cyst fluid is becoming widespread clinically available with the increased availability of NGS and reduced costs.^36,115-117^ Recently, a 22-gene NGS panel (PancreaSeq) was examined in a multicenter cohort of over 1800 patients with pancreatic cysts.^118^ In this study,^118^ the PancreaSeq was not sensitive and specific for various pancreatic cyst types and advanced neoplasia arising from mucinous cysts and, also, had better diagnostic performance than comparative clinical cyst guidelines. In addition, the PancreaSeq also revealed the diversity of genomic alterations seen in pancreatic cysts and their clinical relevance.^118^

Variations in expression of glycosylated, high-molecular-weight glycoproteins, like MUCs have been highlighted as novel biomarkers for early detection of IPMN-associated invasive cancer and differentiation of mucinous from non-mucinous pancreatic cysts.^119^ Other promising biomarker results that may be analysed in cyst fluid, like differentially methylated DNA, telomerase activity, protease expression have not been vigorously validated in diverse cohorts of pancreatic cysts.

Nonetheless, acquiring pancreatic cyst fluid and juices require invasive procedures, challenging operational systems and are highly investigator-dependent. Although some of the aforementioned biomarkers are currently in use in some centres, high costs, reduced availability and variable method sensitivity, advocate for a multimodal approach, rather than identifying a single optimal biomarker.

### Biomarkers in Stool and the Role of Faecal Microbiome in Early Detection of Pancreatic Cancer

In theory, pancreatic juice with exfoliated cancer or pre-cancerous cells harbouring tumour-specific mutations may be secreted into the intestines and hence be discovered as mutations in the stool. This has indeed been investigated in a few studies, as reviewed by Sammallathi et al,^120^ but has yet to make it into clinical practice. Related to this, is the specific microbiome and patterns related to alteration sin healthy compared to patients with cancer.^121-123^ The current field is too premature to allow for any diagnostic or screening measures to be clinically meaningful,^55,124^ but improved understanding of this field may facility better methods in the future. Indeed, a recent study^81^ using 2 cohorts from Spain and Germany demonstrated very promising data using shotgun metagenomic and 16S rRNA amplicon sequencing of fecal microbiota. The microbial pattern together with serum levels of CA 19-9 provided very high accuracy for detection of pancreatic cancer. The study suggests that such specific fecal microbiota-based screening for the early detection of PDAC may become feasible in the future.

### Liquid Biopsies and Circulating Biomarkers

Several metabolic alterations follow the progression of pancreatic cancer.^35,62,125^ Thus, circulating elements that may be derived from precursor lesions or pancreatic cancers are of interest as genomic and proteomic biomarkers,^8,126-128^ as well as circulating cancer cells (CTCs) and exosomes,^58^ and cell free DNA (cfDNA).^129-136^ Such biomarkers have been used to demonstrate the ability to detect several cancer types,^137^ with ability to diagnose at an early stage for when resection is possible.^138^ One such biomarker test called CancerSEEK was designed detect early-stage cancers across anatomical locations (not specific for pancreatic cancer, but including PDAC), through assessment of the levels of circulating proteins and mutations in cell-free DNA^138^ and demonstrated ability for early detection. Others have looked into multi-biomarker panels, adding CA 19-9 to the panel of markers in order to increasing the diagnostic accuracy.^139^ While promising, none have reached a routine clinical implementation as it stands. A large meta-analysis^140^ of all available studies included 19 studies at the time, with some 1872 individuals. The studies were designed to explore liquid biopsies for diagnosis of PDAC.^140^ Seven of the cohorts found were studies on ctDNA, 7 were on CTCs and 6 were investigating exosomes. The overall sensitivity, specificity and AUC of the sROC curve for overall liquid biopsy in detecting PDAC were .80 (95% CI 0.77-.82), .89 (95% CI 0.87-.91) and .95, respectively.^140^ The AUC is not very good and, better diagnostic accuracy is clearly needed.^141,142^ The meta-analysis^140^ confirmed that liquid biopsy had high diagnostic value in detecting PDAC, with exosomes showed highest sensitivity and specificity.^140^ Possibly, such biomarkers and improved technology may have the potential to change early cancer detection in the future, yet further work is needed before implementation as a routine screening or diagnostic tool.

## Conclusion

Several barriers to early detection of PDAC remain but developments in novel technology and new fields of research are providing opportunities for improvement. Some of the reported biomarkers, technology and reported accuracy for detection may see translation routine clinical use if validated and robust data on clinical cohort can confirm the diagnostic performance in average-risk or specific high-risk populations. In order to reduce the number of untimely deaths from this dreaded disease, more effective and specific biomarkers for patients having early‐stage pancreatic cancer is needed. Only then may we experience earlier diagnosis and detection at a curable stage allowing for appropriate surgery and multimodal management.
